# Cystatin SN Upregulation in Patients with Seasonal Allergic Rhinitis

**DOI:** 10.1371/journal.pone.0067057

**Published:** 2013-08-12

**Authors:** Yoshimasa Imoto, Takahiro Tokunaga, Yuri Matsumoto, Yuko Hamada, Mizuho Ono, Takechiyo Yamada, Yumi Ito, Tadao Arinami, Mitsuhiro Okano, Emiko Noguchi, Shigeharu Fujieda

**Affiliations:** 1 Departments of Otorhinolaryngology Head & Neck Surgery, Faculty of Medical Sciences, University of Fukui, Fukui, Japan; 2 Department of Medical Genetics, Faculty of Medicine, University of Tsukuba, Tsukuba, Japan; 3 Departments of Otorhinolaryngology, Graduate School of Medicine, Dentistry and Pharmaceutical Sciences, University of Okayama, Okayama, Japan; 4 Japan Science and Technology Agency, Core Research for Evolutional Science and Technology (CREST), Tokyo, Japan; Ludwig-Maximilians-University Munich, Germany

## Abstract

Seasonal allergic rhinitis (SAR) to the Japanese cedar, *Cryptomeria japonica* (JC) pollen is an IgE-mediated type I allergy affecting nasal mucosa. However, the molecular events underlying its development remain unclear. We sought to identify SAR-associated altered gene expression in nasal epithelial cells during natural exposure to JC pollen. We recruited study participants in 2009 and 2010 and collected nasal epithelial cells between February and April, which is the period of natural pollen dispersion. Fifteen patients with SAR-JC and 13 control subjects were enrolled in 2009, and 17 SAR-JC patients, 13 sensitized asymptomatic subjects (Sensitized), and 15 control subjects were enrolled in 2010. Total RNA was extracted from nasal epithelial cells and 8 SAR-JC patients and 6 control subjects in 2009 were subjected to microarray analysis with the Illumina HumanRef-8 Expression BeadChip platform. Allergen-stimulated histamine release was examined in the peripheral blood basophils isolated from patients with SAR. We identified 32 genes with significantly altered expression during allergen exposure. One of these, *CST1* encodes the cysteine protease inhibitor, cystatin SN. *CST1* expression in nasal epithelial cells was significantly upregulated in both the 2009 and 2010 SAR-JC groups compared with the control groups. Immunohistochemical staining confirmed the increased expression of CST1 in the nasal epithelial cells of SAR patients. Addition of exogenous CST1 to basophils inhibited JC allergen-stimulated histamine release in vitro. We propose that CST1 may contribute to inactivation of protease allergens and help re-establish homeostasis of the nasal membranes.

## Introduction

Allergic diseases such as asthma and allergic rhinitis are major causes of morbidity in developed countries, and are increasing in frequency. In Japan, the prevalence of rhinitis was 16% in 1992 and 21% in 2002 [1]. Seasonal allergic rhinitis (SAR) due to the Japanese cedar (JC) *Cryptomeria japonica*pollen is an IgE-mediated type I allergy that affects the nasal mucosa and causes sneezing, itchiness, and nasal obstruction. It is one of the most common causes of allergy in Japan and is thus a major public health issue [2]. We have recently reported that the prevalence of allergic rhinitis in Japanese adults is 44.2%, and the most common allergen is the JC pollen [3]. Although all patients with SAR are positive for allergen-specific IgE, it is well known that the presence of specific IgE alone is insufficient to develop SAR. Therefore, there is a gap in our understanding of the events that link sensitization to the development of the disease. Two different basic theories on the pathogenesis of allergy have been suggested. One is that allergy is caused by unbalanced and overactive immunological responses against allergens, mostly driven by infiltration of type 2 helper T lymphocytes (Th2), mast cells, and eosinophils into the site of allergic diseases. The other more recent hypothesis is dysregulation of the epithelial barrier, so called “epithelial barrier dysfunction hypothesis,” that is, dysfunction and disruption of the epithelial barrier has been suggested for the primary defects in allergic diseases. Disruption of the skin barrier by filaggrin null mutations has been extensively reported to be associated with the development of atopic dermatitis and other allergic diseases [4], [5]. Recently, Chen et al. reported that Pen c 13, a major allergen secreted by *Penicillium citrinum*, degraded occludin, zonula occludens (ZO)-1, and E-cadherin by nasal Pen c 13 challenge in BALB/c mice, showing increased airway hyperresponsiveness, inflammatory cell infiltration, and mucous overproduction in the lung, as well as increased total IgE and Pen c 13-specific IgE [6]. These data support the notion that dysfunction of epithelial barriers may lead to allergic diseases.

There have been few investigations at the molecular level of the characteristics that distinguish SAR patients from subjects who are allergen-specific IgE-positive, but remain asymptomatic. Such investigations will be essential to identify the molecular and cellular mechanisms by which allergic diseases develop, and they could lead to the discovery of novel preventive therapeutics.

One approach to understanding the molecular basis of allergic inflammation is to identify the differences in the gene expression during natural allergen exposure of patients with varying disease status. Microarray gene expression profiling permits thousands of genes to be analyzed simultaneously. Recently, we used microarray analysis to show the upregulation of interleukin-17 receptor B (*IL17RB*) expression in CD4^+^ T cells of SAR patients during natural allergen exposure [7]. Our study identified fewer genes that were modulated by allergen than those that had been observed in other microarray studies in which CD4^+^ cells were stimulated in vitro, suggesting that changes in the gene expression during natural exposure to allergen in vivo may be subtle. However, it may be more informative to investigate SAR-related gene expression in nasal mucosa rather than in peripheral blood cells, because nasal mucosa is the site of the allergic inflammatory response. Indeed, a microarray analysis of nasal epithelial samples from patients with allergic patients revealed gene expression signatures that were strongly associated with exacerbated or uncontrolled asthma [8], [9]. A recent study using nasal epithelial cells revealed a Th2-driven epithelial phenotype in pediatric patients with house dust mite allergy [8]. Although the entire nasal mucosa and underlying tissues are involved in the development of SAR, we may gain valuable insight into the molecular mechanisms of allergen sensitization and SAR development by comparing gene expression profiles in nasal epithelial cells from healthy subjects, patients with SAR, and sensitized subjects who are positive for allergen-specific IgE, but have not yet developed SAR.

In the present study, we performed a microarray analysis of nasal epithelial cells from SAR patients and Control subjects, and analyzed a candidate gene for RT-PCR using samples of Controls, SAR patients, as well as Sensitized subjects to identify gene signatures that reflect the status of the SAR patient during natural allergen exposure. We observed that the expression of the cysteine protease inhibitor cystatin SN (*CST1*) was upregulated specifically in SAR-JC patients during natural allergen exposure. Our results also showed CST1 may inhibit JC allergen-stimulated histamine release in vitro.

## Methods

### Ethics Statement

All participants provided written informed consent, and the study was approved by the ethics committees of the University of Tsukuba and the University of Fukui, Japan.

### Subjects

Study participants were selected from 1575 hospital workers and university students, who participated in an epidemiological survey on allergic rhinitis between 2003 and 2007. All participants were of Japanese origin and were residents of Fukui prefecture, Japan. The characteristics of the study population have been described in detail elsewhere [3]. Total and allergen-specific IgE (specific for JC, *Dermatophagoides* [dust mites], *Dactylis glomerata* [orchard grass], *Ambrosia artemisiifolia* [ragweed], or the fungal allergens *Candida albicans* and *Aspergillus* spp.) were measured by the ImmunoCAP method (Pharmacia Diagnostics AB, Uppsala, Sweden).

We invited 14 of the 1575 survey subjects to participate in the microarray analysis study. Samples of nasal epithelial mucosa were collected by brushing the inferior turbinate, between February and April 2009—the period of natural exposure to JC pollen. Eight subjects had SAR due to JC pollen (SAR-JC group), which was diagnosed on the basis of a positive history of rhinitis between February and April and high levels of JC-specific serum IgE . None of the SAR patients had detectable IgE specific for *Dermatophagoides* spp., *Dactylisglomerata*, *A. artemisiifolia*, *C. albicans*, or *Aspergillus* spp. and none of the subjects had been treated by oral corticosteroid during pollen season when we collected the samples. They had also not taken intranasal and oral corticosteroids for at least 10 months before the collection of samples. All patients were classified as moderate to severe, according to the allergic rhinitis severity (ARIA) classification [10]. The Control group consisted of 6 subjects for whom the inclusion criteria were as follows: (1) no symptoms or history of allergic diseases, (2) no detectable IgE antibodies specific for the 6 common inhalant allergens, the same as the allergens mentioned above, and (3) total serum IgE levels below the general population mean. We also recruited an additional 7 SAR-JC patients and 7 Controls for real-time RT-PCR analysis.

In 2010, we enrolled an additional samples of 17 SAR-JC patients, 13 asymptomatic subjects with JC-specific IgE (Sensitized group), and 15 Control subjects into the study for RT-PCR validation of the microarray data. Subjects in the Sensitized group and SAR-JC group were negative for IgE specific for *Dermatophagoides* spp., *Dactylisglomerata*, *A. artemisiifolia*, *C. albicans*, or *Aspergillus* spp. The characteristics of the subjects are listed in Table 1.

**Table 1:** Characteristics of subjects for expression analysis.

	SAR-JC	Sensitized	Control	P value
year	2009			
Number of subjects	15	N.A	13	
Male/Female	6/9	N.A	8/5	0.45
Age (mean ± SD)	37.93±9.32	N.A	32.85±7.83	0.13
Total IgE (mean, 95%IC)	44.47 (23.79–83.12)	N.A	36.85 (20.04–67.77)	0.65
Specific IgE (JC) (mean, 95%IC)	8.56 (4.13–17.71)	N.A	<0.34	<0.0001
year	2010			
Number of subjects	17	13	15	
Male/Female	8/9	4/9	6/9	0.64
Age (mean ± SD)	30.47±7.83	24.9±5.75	24.6±5.46	0.24
Total IgE (mean, 95%IC)	76.52 (53.30–109.84)	61.26 (42.54–88.21)	26.00 (12.06–56.07)	0.0084
Specific IgE (JC) (mean, 95%IC)	16.19 (8.26–31.76)	2.97 (1.37–6.43)	<0.34	<0.0001

N.A, not applicable.

### Microarray analysis

Nasal epithelial cells were collected from the 14 subjects in the 2009 by brushing the inferior turbinate with a Cytosoft Cytology Brush (Medical Packaging Co, Camarillo, CA, USA). RNA was extracted using the RNeasy Mini Kit (Qiagen K.K., Tokyo, Japan) according to the manufacturer's instructions. We assessed the quality of total RNA by performing agarose gel electrophoresis and used RNA showing 2 distinct ribosomal peaks corresponding to either 18S or 28S (28S>18S) for microarray and qRT-PCR experiments. Two samples (1 from a SAR patient and 1 from a Control subject) were excluded from the analysis because of the low quality of RNA. Therefore, a total of 12 samples were used for the subsequent microarray analysis.

Microarray assays were performed using the Illumina BeadArray single-color platform (Illumina, San Diego, CA). For the assay, cRNA was synthesized with an Illumina TotalPrep RNA Amplification Kit (Ambion, Austin, TX) according to the manufacturer's instructions. In brief, aliquots of 500 ng of total RNA were reverse transcribed to first- and second-strand cDNA, which was purified by using spin columns, and then transcribed in vitro to yield biotin-labeled cRNA. A total of 750 ng biotin-labeled cRNA was hybridized to each Illumina HumanRef-8 BeadChip array (Illumina) at 55°C for 18 h. The hybridized BeadChip was washed and labeled with streptavidin-Cy3 (GE Healthcare, Waukesha, WI) and then scanned with the Illumina BeadStation 500 System (Illumina). The scanned image was analyzed by using the BeadStudio software (Illumina). In this system, 22,000 transcripts representing 8 whole-genome samples can be analyzed on a single BeadChip. We included at least 1 technical replicate (i.e., the same cRNA sample) for each BeadChip. We used cRNA from the same sample as our technical replicates to determine whether the experiment was performed properly. The correlation coefficients for replicate RNAs were 0.995–0.996 (r^2^). Microarray data was deposited in the Gene Expression Omnibus (www.ncbi.nlm.nih.gov/geo) under references GSE43523.

### Quantitative real-time RT-PCR (qRT-PCR)

RNA was isolated from nasal epithelial cells obtained from the 45 subjects in 2010, as described above. We performed qRT-PCR on RNA by using the TaqMan Universal Master Mix and an Assays-on-Demand Gene Expression Kit (Applied Biosystems, Foster City, CA) according to the manufacturer's instructions. The relative expression was quantified by the ΔΔC_T_ method by using GAPDH as the endogenous control and was performed using SDS software 2.2.0 (Applied Biosystems).

### Immunohistochemical staining

In order to validate the quality of nasal epithelial cells by brushing the inferior turbinate with a Cytosoft Cytology Brush (Medical Packaging Co), nasal epithelial cells from 3 SAR-JCP and 3 control subjects were fixed on glass slides and stained for cytokeratin AE1/AE3 (Dako Corp., Carpinteria, CA) and vimentin (Dako).

Specimens of the inferior turbinate were collected from 6 patient with severe perennial nasal allergy and SAR symptoms (high levels of JC- and dust mite-specific serum IgE) and 5 Control subjects without nasal allergy. Characteristics of subjects for immunohistochemical staining is shown in Table S1. The inferior turbinate specimens were fixed in neutral buffered formalin (10%v/v formalin in water, pH 7.4) and embedded in paraffin wax. Immunohistochemical staining was performed on4-µm-thick sections using a CST1-specific antibody (Abnova Corporation, Taipei, Taiwan), as described previously [11].

### Isolation of basophils and histamine release assay

Peripheral blood mononuclear cells (PBMCs) were isolated with Lymphoprep (Cosmo Bio, Tokyo, Japan) from heparinized whole blood drawn from 6 patients with SAR (3 males and 3 females, aged 24–36 years, high levels of JC-specific serum IgE). Basophils were separated from PBMCs by using a human basophil isolationkit (MiltenyiBiotec GmbH, BergischGladbach, Germany) according to the manufacturer's instructions. Basophils (2×10^4^) were resuspended in RPMI1640 medium (Nissui Pharmaceutical, Tokyo, Japan) supplemented with 100 U/mL penicillin (Sigma-Aldrich, St. Louis, MO), 100 µg/mL streptomycin (Sigma-Aldrich), and 10% fetal calf serum (Thermo Fisher Scientific, Waltham, MA), and aliquoted at 200 µL/well in 96-well microtiter plates. The plates were then incubated at 37°C in a humidified 5% CO_2_ atmosphere for 30 min. The JC pollen allergen Cry j1 (10 µg/mL; Torii, Tokyo, Japan) was preincubated with recombinant CST1 (200 ng/mL) for 30 min at 37°C, then aliquots of this mixture or Cry j1 alone were added to the basophils and the plates were incubated for an additional 30 min. The concentration of histamine released into the supernatant was measured with a Histamine Enzyme Immunoassay kit (BertinPharma, Montigny le Bretonneux, France) according to the manufacturer's instructions.

### Statistical methods

Total and specific IgE levels were expressed as the geometric means with 95% confidence intervals (CI). Total and specific IgE data were log-transformed and statistical analyses of IgE levels and age were performed by Student's *t*-test or ANOVA. Statistical analysis of the gender ratio was evaluated with Fisher's exact test.

For the microarray analysis, background-corrected values for each probe on the BeadChip array were extracted using GenomeStudio software (Illumina). Detection *P*-values were computed from the background model characterized by the chance that the target sequence signal was distinguishable from the negative controls on the same chip. This is based on the average of negative control genes and is termed “the method of background normalization” by Illumina. We performed normexp-by-control background correction, quantile normalization, and log2 transformation on the raw data using the “neqc” command of limma [12]. The normalized values were analyzed by using theR-2.13.1 statistical computing software (R-project, http://www.r-project.org/). Statistical significance of the microarray data was calculated using Welch's t test, and multiple tests were corrected by the Benjamini and Hochberg false discovery rate [13]. A heatmap of genes differentially expressed between SAR patients and Controls was generated using R-2.13.1 statistical computing software (R-project, http://www.r-project.org/). For comparison with the data generated in this study, we extracted the values for the gene expression in eosinophils from the data reported by Nakajima et al [14] (http://www.nch.go.jp/imal/GeneChip/public.htm). The qRT-PCR results were analyzed by Mann-Whitney's U test.

Gene Set Enrichment Analysis (GSEA) was performed using GSEA v2.0.9 [15] to explore the enriched gene sets related to SAR. We performed GSEA using a dataset provided by the GSEA website (c2.v2.symbols.gmt, http://www.broadinstitute.org/gsea/downloads.jsp), as well as with gene sets we manually curated. These sets comprised differentially expressed nasal epithelial genes from children with dust mite allergy (MITE_ALLERGY), genes differentially expressed (2-fold upregulated) during in vitro induction by IL-4 and IL-13 (IL-4_AND_IL_13), and those genes induced by IFNs (2-fold up) (IFNα, IFNβ, and IFNγ) reported by Giovannini-Chami et al. [8]. The manually curated genes are listed in Table S2. The histamine release test results were analyzed using Wilcoxon signed-rank test.

## Results

### Highly expressed genes in nasal mucosa of subjects in the SAR-JC group

**Figure 1:**
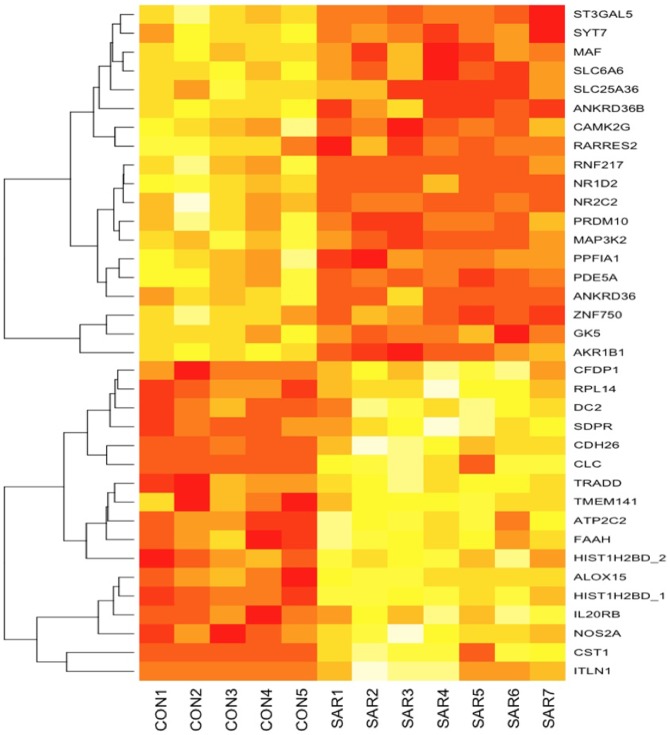
Heatmap of differentially expressed genes between SAR patients (SAR) and Controls (CON).

The immunohistochemical experiment revealed that most cells were positive for cytokeratin, suggesting most of the brushed nasal cells were of epithelial origin (Figure S1). To analyze differences in gene expression between the 7 SAR-JC subjects and 5 Control subjects in 2009, we first selected the transcripts that were expressed in at least 1 of the 12 samples and had detection P values of<0.01 on the BeadChip array. This yielded 13,188 expressed transcripts. Transcripts were considered as upregulated or downregulated if they showed an average of greater than 2-fold increase or decrease in expression, and exhibited a statistically significant difference between the SAR and Control groups (q<0.05). We identified 32 transcripts that were upregulated/downregulated in the nasal mucosa of the SAR subjects by at least 2-fold (q<0.05) (Figure 1 and Table 2). Among them, several genes are known to be strongly expressed in eosinophils [14] (Charcot-Leyden crystal protein [*CLC*] and cadherin-like 26 [CDH26]), and GSEA analysis using the dataset of c2.v2.symbols.gmtrevealed that the microarray dataset in the present study using 7 SAR patients and 5 Controls were enriched for the Top 30 increased eosinophil-specific transcripts (nominal P = 0.006 and FDR q = 0.043), suggesting that their upregulation in the SAR patients may partially be because of the infiltration of eosinophils into the nasal mucosa. GSEA using the gene dataset reported by Giovannini-Chami [8] revealed that genes upregulated in children with mite allergy(MITE ALLERGY) and genes induced by IL-4 and IL-13 (IL-4_AND_IL_13) were enriched in SAR patients in our dataset (Table 3). However, genes related to IFN responses were not enriched in our dataset.

**Table 2:** Genes significantly altered in nasal epithelial cells in SAR-JC patients.

Gene	Array address ID	NM numbers	Fold change	q values**
CST1*	6370541	NM_001898.2	64.6	0.031
ITLN1*	7610170	NM_017625.2	16.9	0.045
CLC	2030142	NM_001828.4	5.9	0.032
CDH26	4590541	NM_021810.3	5.2	0.031
NOS2A*	4760324	NM_153292.1	4.9	0.033
HDC	6280326	NM_002112.2	3.4	0.049
HIST1H2BD	290730	NM_138720.1	3.1	0.016
RPL14	2140753	NM_001034996.1	2.7	0.033
HIST1H2BD	6200669	NM_138720.1	2.5	0.033
OSTC	4610722	NM_021227.2	2.5	0.032
IL20RB*	3400095	NM_144717.2	2.4	0.031
ATP2C2	2510669	NM_014861.2	2.3	0.031
RRAS*	4570670	NM_006270.3	2.2	0.047
CFDP1	6200494	NM_006324.2	2.2	0.033
SDPR*	6290114	NM_004657.4	2.0	0.031
BCL2L15	1710575	NM_001010922.2	2.0	0.045
CAMK2G	1010475	NM_001222.2	−2.0	0.033
ZNF750	3420176	NM_024702.2	−2.1	0.032
SLC6A6	2810280	NM_003043.3	−2.2	0.031
NR1D2	2640707	XM_001130839.1	−2.2	0.031
MAP3K2	3800762	XM_001128799.1	−2.3	0.031
MAF	3610440	NM_005360.3	−2.3	0.033
SLC25A36	3870242	NM_018155.1	−2.3	0.041
GK5	6180543	NM_001039547.1	−2.4	0.041
SYT7*	5050390	NM_004200.2	−2.4	0.031
AKR1B1*	4260139	NM_001628.2	−2.4	0.025
ST3GAL5*	5260403	NM_001042437.1	−2.4	0.031
OTX1*	1740438	NM_014562.2	−2.8	0.049
RORA	1110180	NM_002943.2	−2.9	0.044
PDE5A	1770333	NM_001083.3	−3.1	0.033
ANKRD36*	5810050	NM_198555.3	−4.1	0.044
ANKRD36B	3060010	NM_025190.2	−5.5	0.031

* Absent call in Eosinophils according to the data by Nakajima et al.

** Welch's t test, Benjamini-Hochberg FDR.

**Table 3:** GSEA using dataset of the study by Giovannini-Chami [5].

	Enrishment Score (ES)	Nominal ES (NES)	P value	q value
IL-4_AND_IL_13	0.73	1.85	0.004	0.027
MITE_ALLERGY	0.87	1.75	0.004	0.025
IFN α	0.21	0.74	0.78	0.89
IFN γ	0.19	0.66	0.83	0.75

**Figure 2:**
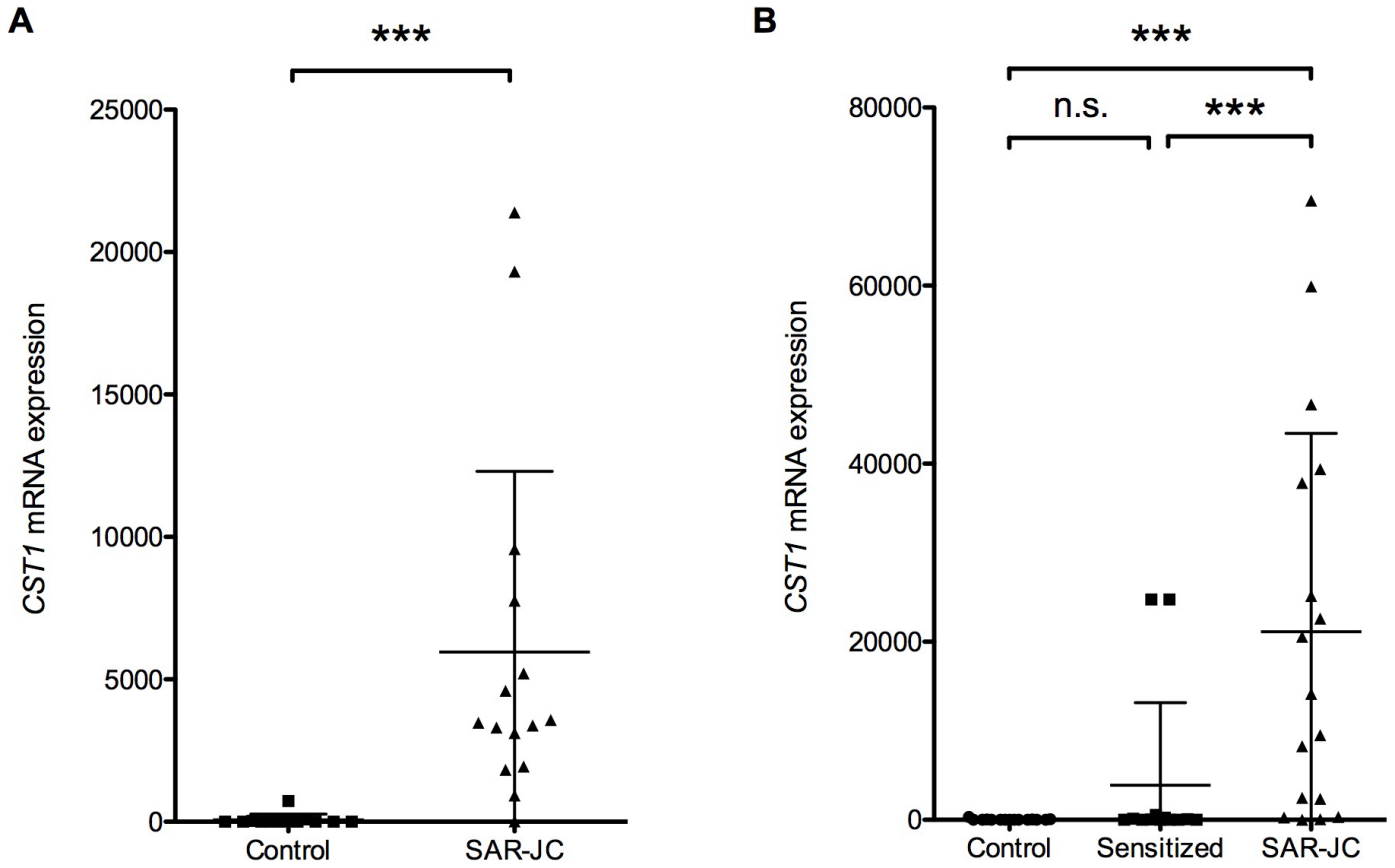
Expression of *CST1* in nasal epithelial cells. RNA from nasal mucosa of Control subjects, Sensitized subjects, and patients with SAR-JC was analyzed for *CST1* expression by qRT-PCR. Expression of *CST1* in samples from (A) 2009 and (B) 2010. ns, not significant (P>0.05); ***, P<0.001.

We found that *CST1* (cystatin SN) was highly expressed in the SAR-JC subjects, undetectable in the Control subjects, and not expressed in eosinophils (absent call judged by the GeneChip Analysis Suite 5.0 program according to the data by Nakajima et al [14]; hence, we focused on *CST1* for further analysis. To validate the results of the microarray analysis, we measured *CST1* expression in the nasal mucosal samples from the 2009 subjects by qRT-PCR. As can be seen in Figure 2A, *CST1* expression was significantly higher in the SAR-JC group than in the Control group (P = 2.4×10^−7^). To confirm these data, we also performed *CST1* qRT-PCR on nasal mucosal samples in 2010 (Figure 2B). Here, *CST1* expression was significantly higher in the SAR-JC group than in the Sensitized group (P = 0.00087) and Control group (P = 0.0006), whereas there was no significant difference in expression between the Sensitized and Control groups (P = 0.13).

**Figure 3:**
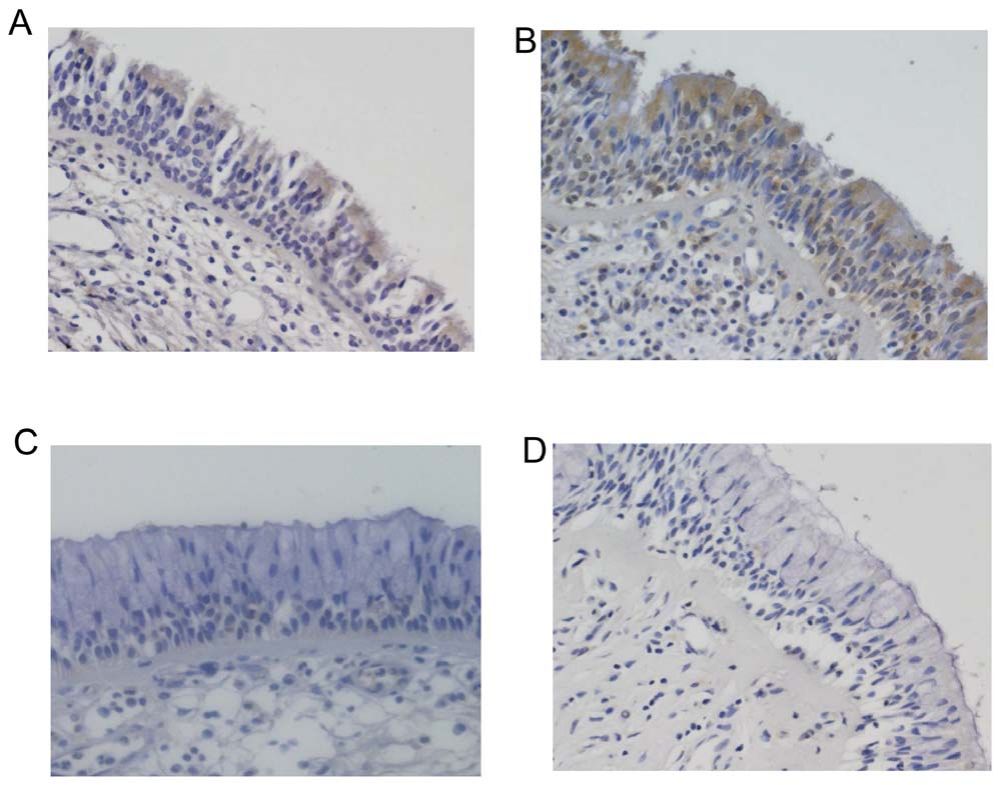
Immunohistochemical staining of CST1 in nasal epithelial cells. Representative immunostaining of CST1 expression inthe nasal epithelium of 2 SAR patients (A and B) and 2 Control subjects (C and D). Magnification:×200.

**Figure 4:**
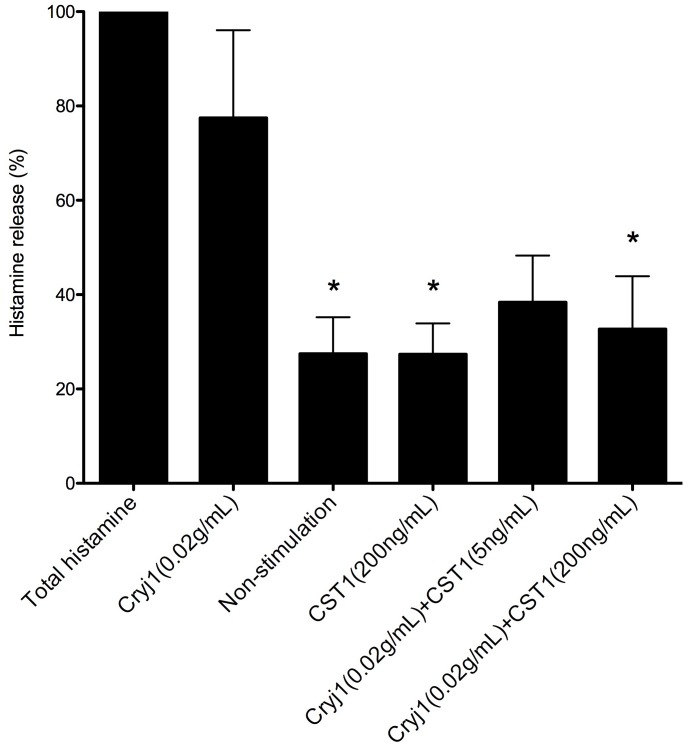
Histamine release by peripheral basophils stimulated with JC allergen. Cells were cultured with Cryj1 both in the presence and absence of recombinant CST1. *, P<0.05.

We next investigated the expression of CST1 protein by immunohistochemical staining of the inferior turbinate samples from6 patients with perennial nasal allergy and SAR symptoms and 5 Control subjects without allergy. As shown in Figure 3, CST1 was expressed in nasal epithelial cells in the SAR patients, but not in the Control subjects. To examine the effect of CST1 on allergen-stimulated histamine release, basophils were isolated from the peripheral blood of 6 patients with SAR, and stimulated with the JC allergen Cry j 1 both in the presence and absence of CST1. Figure 4 shows that Cry j 1-stimulated histamine release by basophils was effectively inhibited in the presence of CST1 (P = 0.032).

## Discussion

In the present study, we performed microarray analyses to identify SAR-related genes in nasal epithelial cells. We identified 36 genes that showed statistically significantly differed in SAR subjects compared with the Control group, and we confirmed by qRT-PCR that the expression of *CST1* was upregulated in SAR-JC patient compared with the Control group in both 2009 and 2010. We also showed that addition of exogenous CST1 to basophils inhibited JC allergen-stimulated histamine release in vitro.

Cry j1 is a major antigen of Japanese cedar pollen [16]. It is a basic glycoprotein localized in the pollen cell wall, which has pectate lyase activity [17]. The epitope of Cry j1 is recognized by specific IgE of Cry j1 and released histamine from basophils by interacting antigen and antigen-specific IgE. Alteration of Cry j1 structure by heat denaturation leads to reduction of IgE-binding activities [18], [19]. Therefore, histamine release from the basophils of patients induced by adding Cry j1 might be due to the degranulation via crosslinking of Cry j1-specific IgE already bound on the IgE receptor. Takai et al. reported that interaction of Der f 1, one of the major allergen of house dust mite with protease activity, and cystatin A, which belongs to cystatin superfamily, inhibited the binding of IgE in mite-allergic volunteers' sera, suggesting that cystatin A might change the binding site between antigen and antigen-specific IgE [20]. Thus, cystatin SN might affect the binding site between antigen and antigen-specific IgE, leading to the suppression of histamine release from basophils in the present study.

Cysteine proteases are widely expressed proteolytic enzymes that play roles in inflammatory tissue destruction as well as tissue remodeling [21]. The proteolytic activity of these enzymes is controlled by a family of inhibitors known as the cystatin superfamily [22], and cystatins inhibit cysteine proteases by forming tight but reversible complexes with their target enzymes. Members of the type 2 cystatin subfamily are widely distributed [23], and include*CST1*, *CST2*, *CST3*, *CST4*, *CST5*, *CSTP1*,and *CSTP2*, which share >70% sequence similarity [24]. The *CST1* is expressed in the submandibular gland, gall bladder, and uterus [24]. CST1 has been shown to bind tightly to the cysteine protease, papain, which is a potent allegen, and to inhibit the cysteine protease activity of papain [25].

Airborne allergens, such as those derived from pollen, cockroaches, fungi, and house dust mites, are proteases with the capacity to induce an inflammatory response. Previous studies have shown that the proteolytic activity of these allergens may facilitate sensitization, possibly by disrupting the balance between protease and antiprotease activities. This theory is supported by the observation that the protease allergens reduce the antiprotease activity of the mucosa [26], [27], [28], [29]. Der p 1 and Der f 1, the major allergens derived from house dust mites, are cysteine proteases that degrade α1-antitrypsin inhibitor in the airway [28], [29] and inactivate lung surfactant proteins A and D [26]. Cystatin A, a member of the cysteine protease inhibitor family, has been shown to suppress induction of Der f 1-specific IgE by modifying the interaction between Der f 1 and cystatin A, which may modulate the allergic inflammation and sensitization processes [20]. Thus, the cystatin family may play protective roles against allergens with protease activity.

Measurement of allergen-specific IgE in serum is widely used to diagnose allergic diseases such as SAR and asthma; however, subjects with allergen-specific IgE do not necessarily develop the allergic disease. Although many reports have described the pathophysiological features of allergic inflammation, biomarkers or genes that predict the onset of allergic rhinitis are yet to be reported. In this study, we observed that *CST1* was not expressed in cells from non-sensitized subjects, and that *CST1* expression was significantly lower in the IgE-positive Sensitized subjects than in the SAR-JC subjects. These data suggest that *CST1* may be a candidate marker gene to predict allergic rhinitis. We detected that 2 subjects in the Sensitized group showed particularly high *CST1* expression in the nasal mucosa, and we speculate that these subjects may have been close to developing SAR symptoms.

A recent study using nasal epithelial cells from children with dust mite allergy showed that *CST1* was the most highly upregulated gene and that *CST1* was also strongly induced by stimulation with IL-4 and IL-13 [8]. Therefore, *CST1* is likely to be upregulated in response to protease-containing allergens and the Th2 environment in the nasal mucosa. Because GSEA revealed that the genes upregulated in nasal brushing samples from mite allergic patients and the genes induced by IL-4 and IL-13 were significantly enriched in our dataset (Table 3), our data support the notion that pathways underlying allergic rhinitis are common regardless of the allergen.

The balance between protease and antiprotease activities might thus be a key to distinguish between the sensitization stage and allergic response, and we suggest that cystatins contributes re-establishing homeostasis of the nasal mucosa.

## Supporting Information

Figure S1 **Immunohistochemical staining of cytokeratin and vimentin in brushed nasal epithelial cells.** Representative immunostaining of cytokeratin expression (A and B) and vimentin expression (C and D). Magnification: ×200.
(PPTX)

Table S1 **Characteristics of subjects for immunohistochemical staining.**
(XLSX)

Table S2 **Manually curated gene sets used for GSEA.**
(XLSX)
