# Supplementary figures and images for: Cystatin SN Upregulation in Patients with Seasonal Allergic Rhinitis
Source: PLoS One. 2013 Aug 12;8(8):e67057. doi: 10.1371/journal.pone.0067057 (PMC3741298; doi:10.1371/journal.pone.0067057)

## Slide 1
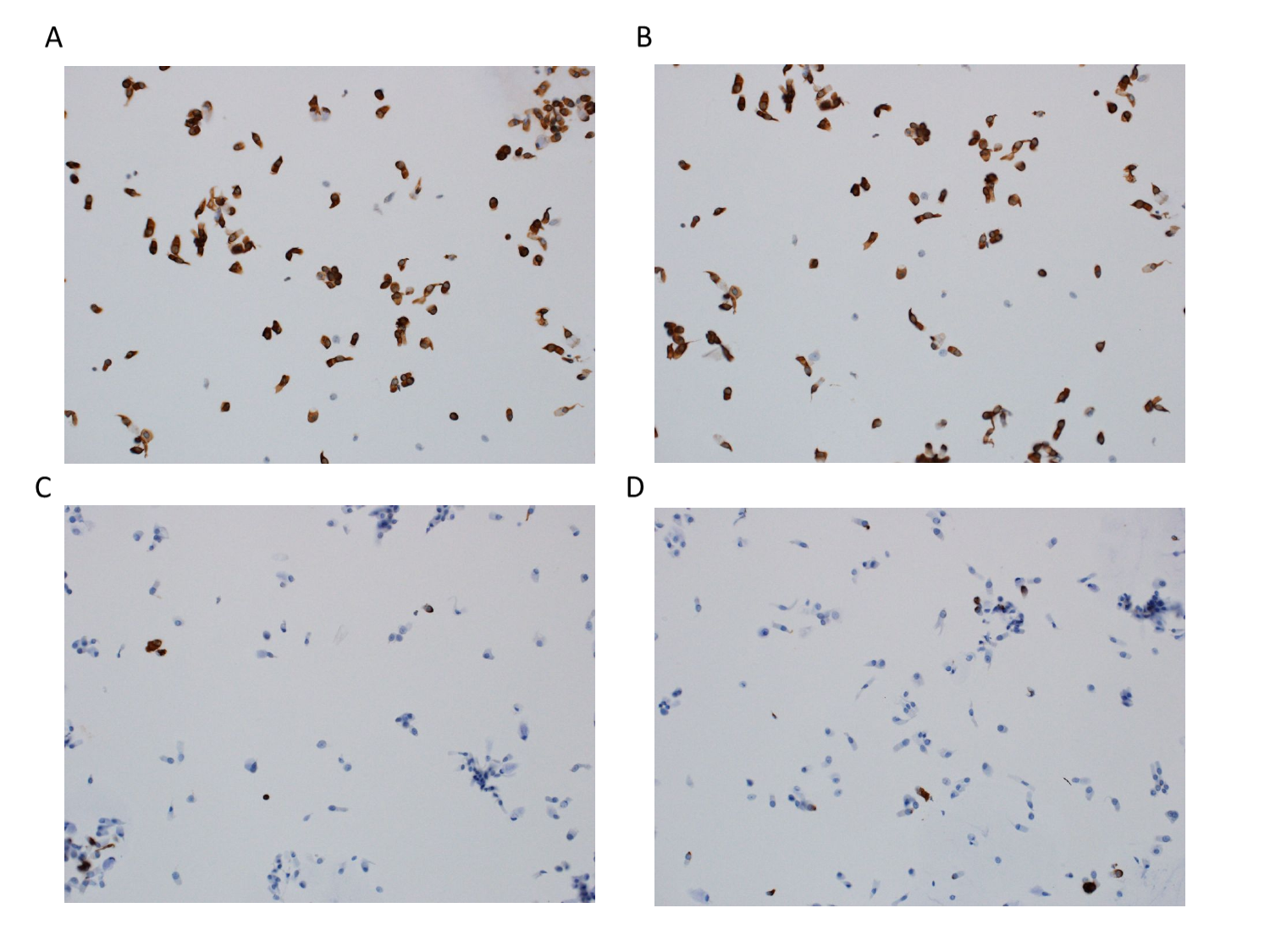

Supplement: Figure S1 — Immunohistochemical staining of cytokeratin and vimentin in brushed nasal epithelial cells. Representative immunostaining of cytokeratin expression (A and B) and vimentin expression (C and D). Magnification: ×200. (PPTX) [file pone.0067057.s001.pptx]
